# Enhanced immunogenicity of the *Brucella* A19 Δ*btpB* mutant leads to accelerated clearance in the host

**DOI:** 10.3389/fimmu.2026.1747903

**Published:** 2026-01-30

**Authors:** Ye Yuan, Xuan Bu, Yunyi Zhai, Gaowa WuDong, Qing Lu, Ting Tang, Dong Zhou, Wei Liu, Yaping Jin, Aihua Wang

**Affiliations:** 1 College of Veterinary Medicine, Northwest A&F University, Yangling, China; 2 Key Laboratory of Animal Biotechnology of the Ministry of Agriculture, Northwest A&F University, Yangling, China; 3 Engineering Research Center of Efficient New Vaccines for Animals, Ministry of Education, Yangling, China

**Keywords:** *Brucella*, BtpB, immune evasion, immunogenicity, vaccine

## Abstract

**Introduction:**

*Brucella* is a Gram-negative facultative intracellular bacterium that can cause fever, abortion, and other symptoms in humans and various mammals. *btpB*, a type IV secretion system (T4SS) effector of *Brucella*, plays a critical role in regulating *Brucella* infection and inhibiting the host's innate immune response.

**Methods:**

In this study, a *btpB* mutant strain of *Brucella* A19 (Δ*btpB*) was constructed using homologous recombination, and its biological characteristics, virulence, and immunogenicity were systematically investigated.

**Results:**

The results showed that Δ*btpB* exhibited weakened resistance to in vitro stress, while its growth characteristics did not differ significantly from the wild-type strain A19. In the mouse immunization model, Δ*btpB* induced weaker splenic pathological damage, and the splenic bacterial load was significantly lower than that of A19, indicating its reduced virulence. Additionally, Δ*btpB* infection elicited stronger humoral and cellular immune responses in mice, including higher antibody levels, increased levels of Th1 cytokines (such as IFN-γ and IL-2), and enhanced proliferation and activity of CD8+ cells. Detection of Th1 and Th2 cells revealed that Δ*btpB* induced stronger Th1 and Th2 responses in the spleen in the early stage, but the Th2 response weakened in the middle and late stages of infection. Notably, Δ*btpB* infection did not suppress natural killer (NK) cell activity and even significantly enhanced its cytotoxic activity compared to the A19 strain.

**Conclusion:**

Our research demonstrates that Δ*btpB* leads to a reduced survival capacity of *Brucella*, while enhancing its immunogenicity. This suggests *btpB* is an important target for the prevention of *Brucella*.

## Highlights

- Δ*btpB* mutant exhibited unchanged adhesion and proliferation capabilities in macrophages, but was cleared more rapidly from the mouse spleen.
- Δ*btpB* induced a stronger Th1 immune response, whereas the level of the Th2 response did not change significantly.
- Δ*btpB* infection led to an increased level of cytotoxic T cells in the mouse spleen.
- Δ*btpB* infection increased the level of Th1 cells in the mouse spleen, whereas the Th2 cell level showed an initial increase followed by a slight decline.
- Δ*btpB* infection resulted in elevated levels and a significantly enhanced activation status of NK cells in the mouse spleen.

## 1 Introduction

*Brucella* is a gram-negative, facultative intracellular parasitic bacterium capable of infecting humans, livestock (cattle, sheep, pigs, camels, etc.), and wild animals (deer, bison, etc.). It typically causes abortion, infertility, and arthritis, leading to significant economic losses and public health risks (1–3). Currently, there are no human vaccines against *Brucella*, while the animal vaccines are all attenuated live strains (*Brucella* Rev.1, *Brucella* A19, *Brucella* RB51). Although these attenuated live vaccines have demonstrated good efficacy through long-term use, they still present certain drawbacks, such as safety risks and interference with serological diagnosis (4–7). Therefore, safer and more ideal vaccines remain essential for brucellosis control. Current research directions for novel *Brucella* vaccines focus primarily on safer subunit vaccines and genetically engineered deletion vaccines based on existing vaccine strains (8–10). A deeper understanding of the roles of *Brucella* components after they enter the host and the functions of various immune cell subsets during infection is essential for guiding the development of novel *Brucella* vaccines.

The control of *Brucella* relies on cell-mediated immunity, which primarily involves activated antigen-presenting cells (macrophages, dendritic cells), CD4^+^, and CD8^+^ T lymphocytes. *Brucella* antigens induce the production of T helper 1 (Th1) cytokines, making the Th1 immune response crucial for eliminating *Brucella* infections (11–13). The immune evasion mechanisms or immunosuppression of the host immune system by *Brucella* have been well-studied. In the early stage of infection, *Brucella* can kill neutrophils, thereby inducing macrophages to phagocytose the dead cells, facilitating their rapid intracellular entry and subsequent proliferation (14, 15). Once inside macrophages, *Brucella* can reduce phagocytic activity and increase autophagy levels, while interfering with the antigen-presenting function of MHC-II molecules to specific T cells (16, 17). Additionally, studies have shown that *Brucella* can impair the maturation of dendritic cells (DCs), thereby affecting their antigen-presenting ability, which in turn impacts the effectiveness of the adaptive immune response (18–20). Wang et al. observed the dysregulation of Th1 responses and exhaustion of CD8^+^ T cells and NK cells in peripheral blood lymphocytes of chronic brucellosis patients (21). Moreover, some studies have also found a significant increase in the proportion of regulatory T cells (Tregs) in infected tissues (22, 23). These findings indicate that *Brucella* has evolved various immune escape and immunosuppressive capabilities against different host immune cells.

BtpB is a Toll/Interleukin-1 Receptor (TIR) domain protein in *Brucella* and an important effector of the T4SS. *Brucella* TIR domain proteins, including BtpA and BtpB, are known to disrupt Toll-like receptor (TLR) signaling in the host, thereby suppressing innate immunity (24, 25). Recent studies have revealed that both BtpA and BtpB modulate host cell energy metabolism by hydrolyzing NAD^+^, leading to the depletion of NADH and ATP. BtpB specifically regulates PDIA4 expression to alter NAD^+^/NADH levels in macrophages (25, 26). In addition, our previous studies indicated that, during *Brucella* infection of macrophages, BtpB inhibits autophagy by reducing the formation of autophagolysosomes, suppresses the expression of TLR2 and TLR4 in macrophages, and weakens the activation of the NLRP3 inflammasome, thereby suppressing the host immune response during *Brucella* infection (27, 28). Suzana P. Salcedo et al. reported that BtpB impairs DC activation by inhibiting the expression of MHC-II molecules on the surface of murine dendritic cells (DCs), and disrupts NF-κB nuclear translocation, suggesting its correlation with the host inflammatory response and antigen presentation (24, 29). In summary, BtpB can induce immunosuppression in the host during *Brucella* infection.

BtpB has been demonstrated to exert significant immunosuppressive effects on both macrophages and dendritic cells (24, 28). Therefore, this study utilized homologous recombination to construct a markerless Δ*btpB* strain. The biological characteristics of the mutants were assessed by observing their growth patterns and *in vitro* stress resistance. Murine immunization experiments were then conducted to evaluate virulence through measurements of the splenic bacterial load and pathological lesions in the spleen and liver. Immune efficacy was further analyzed by detecting the serum antibody levels, relative cytokines, and proliferation of various immune cell populations. These investigations aimed to explore how BtpB deletion alters host immune responses triggered by *Brucella* and to evaluate the potential of Δ*btpB* as a candidate vaccine against brucellosis.

## 2 Materials and methods

### 2.1 Biosafety statement

All experiments were conducted in compliance with the Regulations on Biosafety Management of Pathogenic Microorganism Laboratories (No. 424, 2004, promulgated by the State Council of the People’s Republic of China) and approved by the Biosafety Committee of Northwest A&F University. All animal experiments followed the Guidelines for the Ethical Use and Welfare of Experimental Animals and were reviewed and approved by the Institutional Animal Ethics and Use Committee.

### 2.2 Bacteria, cells, and culture media

The *Brucella abortus* A19 strain was obtained from the Shaanxi Provincial Institute of Veterinary Drug Control (Xi’an, China). It was cultured in Tryptic Soy Broth (TSB, Sigma-Aldrich, St. Louis, MO, USA) or on Tryptic Soy Agar (TSA, Sigma-Aldrich, St. Louis, MO, USA). For liquid culture TSB, *Brucella* A19 cultures in the logarithmic growth phase (OD_600_ ≈ 0.6–0.8) should be subcultured into fresh TSB at an inoculum of 1%–2% for continued growth. Liquid cultures of *Brucella* A19 in the logarithmic growth phase can be streaked or spread onto fresh TSA plates to obtain single colonies. All *Brucella* cultures were incubated at 37 °C under 5%–10% CO_2_. Liquid cultures require shaking (180 rpm) for 48–72 hours, whereas solid plate cultures are incubated statically for 3–5 days until small, transparent, smooth-edged colonies appear. All culture and handling procedures for *Brucella* are conducted under biosafety level 3 laboratory conditions.

RAW264.7 mouse macrophages were maintained in our laboratory and cultured in high glucose DMEM cell culture medium (L110KJ, Basal Media Technologies), supplemented with 10% fetal bovine serum (ZETA LIFE), in a 5% CO_2_ environment at 37 °C.

### 2.3 Construction of the *Brucella btpB* mutant strain

We constructed a *Brucella btpB* mutant strain on the basis of the pK18mobSacB plasmid (Hongsai Biotechnology Co., Ltd) via the homologous recombination method, following a previously described protocol. The plasmid, which carried homologous arms flanking the *btpB* gene, was transformed into *Brucella* cells via electroporation via a Gene Pulser system (Bio-Rad). The transformants were subjected to two rounds of selection: first on TSA media supplemented with 25 μg/mL kanamycin (YuanYe Bio-Tech) and subsequently on TSA medium containing 5% sucrose (YuanYe Bio-Tech). Single colonies were picked and cultured in TSB broth. After confirming BtpB deletion via RT-qPCR, the mutant strain was passaged for 15 generations, and bacterial suspensions were collected for PCR identification to assess genetic stability.

### 2.4 Assay of growth kinetics and stress resistance

We examined the alterations in the *in vitro* stress resistance of Δ*btpB* under acidic, thermal, oxidative, hyperosmotic, and polymyxin B (YuanYe Bio-Tech) conditions. A total of 1×10^6^ colony-forming units (CFU) of A19 and Δ*btpB* were treated in TSB media adjusted to pH 5.4, 42°C, 1 mM H_2_O_2_, 2 M sorbitol and 200 μg/mL polymyxin B for 2 hours. Then, plate counting of the bacterial samples was carried out via 10-fold serial dilutions. Survival rates under the five stress conditions were calculated by comparing CFU counts before and after treatment.

In addition, we evaluated the growth curve of Δ*btpB*. A total of 1×10^9^ CFU of A19 and Δ*btpB* were added to 500mL of TSB medium and then cultured in an incubator shaker. Bacterial samples were collected after 6 h, 12 h, 18 h, 24 h, 30 h, 36 h, 48 h, 60 h, 72 h, and 96 h, and 10-fold serial dilutions was used for plate counting.

### 2.5 Cell adhesion and Intracellular proliferation assays

We first performed cell adhesion assays of Δ*btpB*. Mouse RAW264.7 macrophages were seeded at 5×10^5^ cells/well in 24-well plates and cultured until fully adherent. The cells were infected with A19 or Δ*btpB* at a multiplicity of infection (MOI) of 1:40 and incubated in a cell culture incubator. At 0.5, 1, 2, and 4 hours post-infection, the cells were lysed, and bacterial counts were determined via 10-fold serial dilutions followed by plating for CFU enumeration.

Subsequently, an intracellular proliferation assay was conducted. RAW264.7 cells were seeded and cultured as previously described. The cells were infected with A19 or Δ*btpB* at a MOI of 1:100 and incubated in a cell culture incubator. At 4 hours post-infection, the cells were treated with 1 mL of DMEM containing 50 μg/mL gentamicin (YuanYe Bio-Tech) for 1 hour to kill the extracellular bacteria, and then further cultured with fresh medium containing 50 μg/mL gentamicin. The cells were lysed at 0, 6, 12, 24, 48, and 72 hours post-treatment, and the bacterial counts were determined via 10-fold serial dilution and plating.

### 2.6 Mouse immunization

Female BALB/c mice (6 weeks old) were obtained from Chengdu Dossy Experimental Animal Co., Ltd. After a one-week acclimation period, the mice were divided into three groups and inoculated intraperitoneally with 100 μL of sterile PBS, PBS containing 1×10_7_ CFU of A19, or PBS containing 1×10_7_ CFU of Δ*btpB*. At 1, 2, 4, and 6 weeks post-infection, five mice from each group were weighed, anesthetized, and euthanized by cervical dislocation. Blood samples were collected, and the spleens and livers were dissected. Spleen weights were measured for further analysis. In addition, the body weights of the mice were measured at multiple time points within 42 days post-infection (3, 7, 14, 21, 28, 35, and 42 dpi), and the weight gain relative to that at 0 wpi was calculated.

### 2.7 Histopathological examination and splenic bacterial load quantification

The excised spleens and livers were sectioned, and portions were fixed in 4% paraformaldehyde (MiShushengwu). Fixed tissues were embedded in paraffin, sectioned, and stained with hematoxylin-eosin (HE). The stained sections were observed and photographed under a microscope.

Spleens were weighed after dissection, placed in sterile PBS, and ground into a homogenate via a tissue grinder. The homogenate was serially diluted 10-fold and plated for CFU counting.

### 2.8 Assessment of serum antibodies and cytokine levels in immunized mice

Serum antibody and cytokine levels were detected using ELISA kits (Fankew) according to the manufacturer’s instructions. The optical density (OD) of each sample was measured at 450 nm via a microplate reader (Tecan). Standard curves were generated based on the OD values of the kit-provided standards, and the concentrations of the samples were calculated accordingly.

For *Brucella*-specific IgG detection, ELISA plates were coated with 100 μL of *Brucella* cell lysate (OD_600_≈0.3) as the antigen. After blocking with 5% skim milk, 100 μL of collected serum (primary antibody) was added to the wells, followed by incubation with 50 μL of horseradish peroxidase (HRP)-conjugated goat anti-mouse IgG secondary antibody (Beyotime) at a 1:5,000 dilution. OD values at 450 nm were measured via a microplate reader.

### 2.9 Analysis of splenic lymphocytes in mice

Mouse spleens were homogenized in sterile PBS, and lymphocytes were isolated via a murine lymphocyte extraction kit (Beyotime). Approximately 1×10^6^ lymphocytes were resuspended in 100 μL of sterile PBS. The first batch of cells was stained in light-protected conditions with 3 μL each of PE-conjugated anti-CD3, APC-conjugated anti-CD4, FITC-conjugated anti-CD8, and PerCP-conjugated anti-CD107a antibodies (BioLegend) to assess the level of CD4^+^ and CD8^+^ T-cell proliferation and CD8^+^ T-cell degranulation. The second batch of cells was stained in light-protected conditions with 3 μL each of PE-conjugated anti-CD3, FITC-conjugated anti-CD49b, and PerCP-conjugated anti-CD107a antibodies (BioLegend) to evaluate NK cell proliferation and degranulation.

The remaining lymphocytes were used to analyze Th1/Th2 cell responses. The cells were counted and seeded at 1×10^7^ cells/well in 24-well plates. Heat-killed *Brucella* antigen (HKBA) was added at an MOI of 1:100 as a stimulant, and the cells were incubated for 48 hours. Monensin (1 μL, BioLegend), a protein transport inhibitor, was added 6–8 hours prior to staining. After stimulation, the cells were surface-stained in light-protected conditions with 3 μL each of PE-conjugated anti-CD3 and FITC-conjugated anti-CD4 antibodies (BioLegend) for 30 minutes on ice. Following centrifugation and washing, the cells were permeabilized with fixation/permeabilization buffer (BioLegend) and intracellularly stained with 3 μL each of APC-conjugated anti-IFN-γ and PE-Cy7-conjugated anti-IL-4 antibodies (BioLegend) for 20 minutes at room temperature. Finally, the stained lymphocyte suspensions were analyzed via a flow cytometer (Agilent).

### 2.10 Statistical analysis

All experiments were performed in at least three independent replicates. Statistical analysis was performed via SPSS software. Comparisons between two groups were analyzed via a two-tailed Student’s t-tests, whereas one-way ANOVA was applied for significance assessment among three or more groups. Statistical significance was set at *p* < 0.05, with asterisks (*) indicating significant differences. Graphical presentations were generated using GraphPad Prism software.

## 3 Results

### 3.1 The *btpB* mutant strain presented unaltered growth characteristics but reduced *in vitro* stress resistance compared to A19

**Figure 1:**
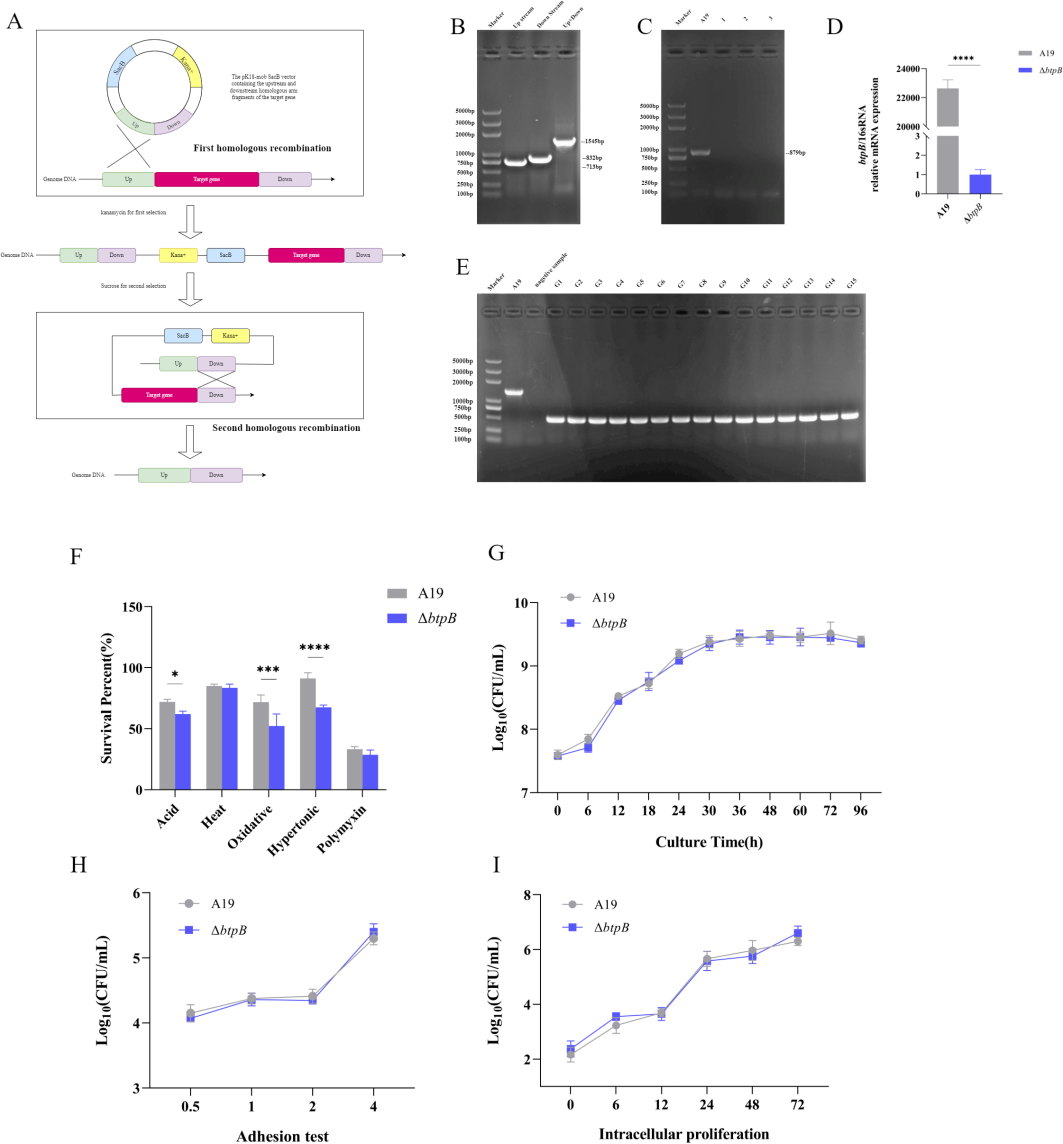
Construction of the *Brucella btpB* mutant strain and analysis of its stress resistance and growth characteristics. **(A)** Schematic diagram of the markerless knockout strategy. **(B)** Amplification of upstream/downstream homologous arms of the *btpB* gene and assembly of the fusion fragment. **(C)** PCR verification of the *btpB* mutant strain. Lanes 1–3: Selected BtpB deletion mutants. **(D)** RT-qPCR analysis of *btpB* gene expression in the mutant strain. **(E)** Genetic stability assessment of the mutant strain after 15 generations of serial passaging. Lanes G1–G15: PCR results from generations 1 to 15. F. Survival rates of A19 and Δ*btpB* under acidic (pH 5.4), thermal (42 °C), oxidative (1 mM H_2_O_2_), hyperosmotic (2 M sorbitol), and polymyxin B (200 μg/mL) conditions. **(G)***In vitro* growth curves of A19 and Δ*btpB* over 96 hours. **(H)** Adhesion capacity of A19 and Δ*btpB* to RAW 264.7 macrophages. **(I)** Intracellular proliferation of A19 and Δ*btpB* in RAW 264.7 macrophages. All the data are represented as the mean ± SEMs of at least three independent experiments. *p < 0.05; ***p < 0.001; ****p <  0.0001.

Using a markerless knockout method (**Figure 1A**), we successfully constructed the Δ*btpB* strain. The homologous arms flanking the *btpB* gene were amplified and cloned, and inserted into the pK18mobSacB plasmid (**Figure 1B**). Following electroporation and two rounds of selection, PCR and RT-qPCR confirmed the successful deletion of *btpB* (**Figures 1C, D**). Serial passaging demonstrated stable genetic inheritance of the deletion (**Figure 1E**).

We evaluated the survival capacity of Δ*btpB* under simulated stress conditions, including acidic (pH 5.4), thermal (42 °C), oxidative (1 mM H_2_O_2_), hyperosmotic (2 M sorbitol), and polymyxin B (200 μg/mL) challenges. The results revealed that Δ*btpB* displayed significantly impaired resistance to acidic, oxidative, and hyperosmotic stresses (**Figure 1F**). After 2 hours of acidic exposure, approximately 70% of the A19 strains survived, whereas 60% of the Δ*btpB* strains survived. Under oxidative stress, survival rates drecreased sharply from 70% (A19) to 50% (Δ*btpB*). Hyperosmotic stress caused minimal effect on A19 (90% survival), but Δ*btpB* survival decreased to 70%. However, BtpB deletion had no significant effect on resistance to thermal stress or polymyxin B (**Figure 1F**).

Subsequent analyses of growth kinetics *in vitro* and cellular infection models showed no significant differences between the strains. *In vitro* growth curves over 96 hours confirmed comparable proliferation rates for A19 and Δ*btpB* (**Figure 1G**). Similarly, cell adhesion and intracellular proliferation assays in macrophages revealed no alterations between the strains (**Figures 1H, I**).

### 3.2 The *btpB* mutant strain was cleared more rapidly in mouse spleens and pathological responses were attenuated.

**Figure 2:**
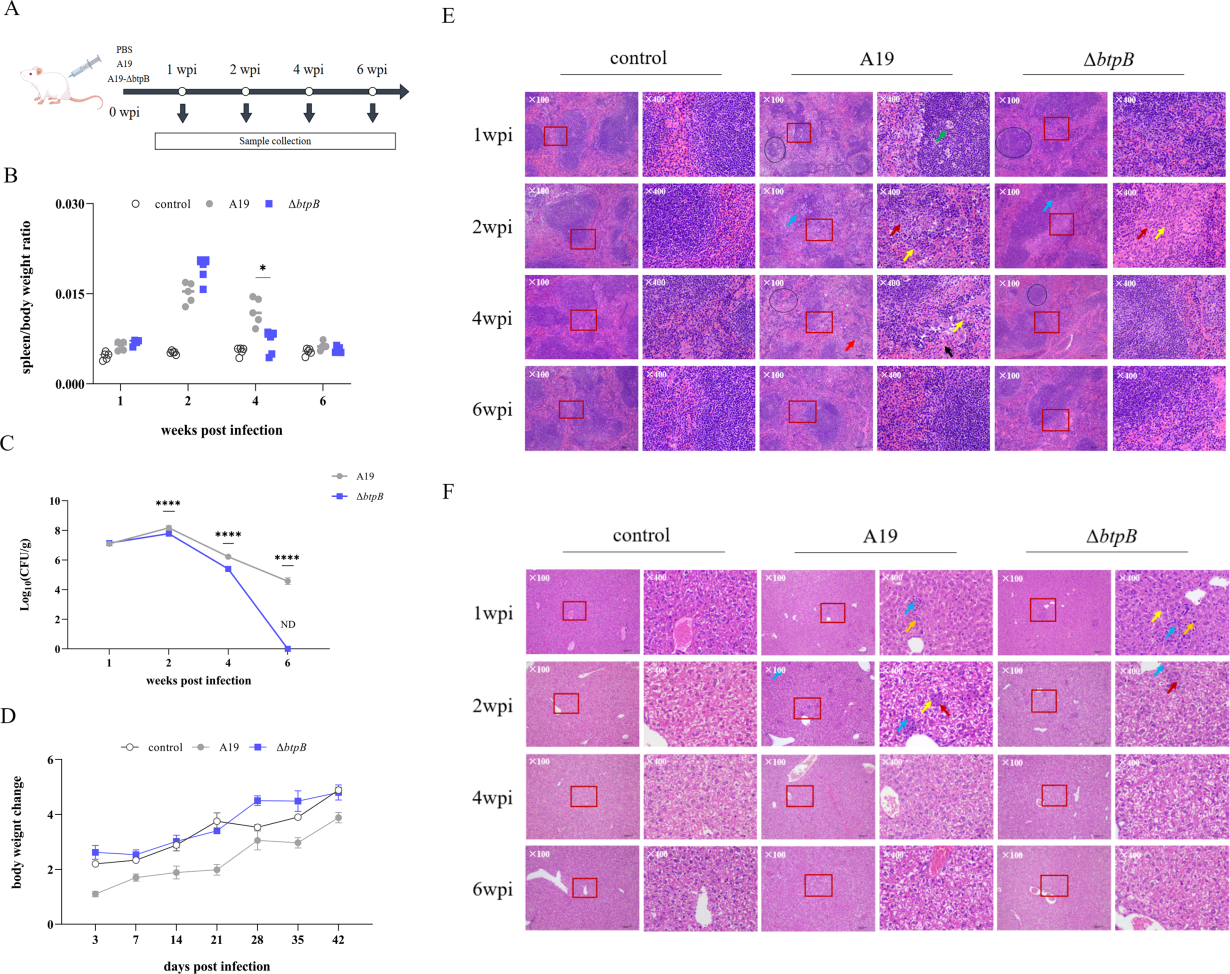
Splenic, hepatic and body weight changes in mice infected with *Brucella* A19 and Δ*btpB*. **(A)** Mouse infection experiment. **(B)** The ratio of spleen/body weight changes at 1, 2, 4, and 6 wpi. **(C)** Splenic bacterial load (CFU/g) at 1, 2, 4, and 6 wpi. **(D)** Body weight gain (g) in mice within 42 days post-infection. **(E)** Histopathological changes in the spleen at 1, 2, 4, and 6 wpi. ↑ Lymphocyte degeneration and necrosis; ↑ Splenic nodule degeneration and necrosis; ↑ Fibrous tissue hyperplasia; ↑ Neutrophils; ↑ Sinus congestion; ○ Disruption of white pulp structure. **(F)** Histopathological changes in the liver at 1, 2, 4, and 6 wpi. ↑ Focal hepatocyte necrosis; ↑ Fibrous tissue hyperplasia; ↑ Neutrophils; ↑ Lymphocyte proliferation. All the data are represented as the mean ± SEMs of at least four independent experiments. *p < 0.05; ****p <  0.0001

Based on the established protocol (**Figure 2A**), we performed a mouse bacterial challenge experiment, collected samples, and detected the virulence and immune indices of *ΔbtpB*. The ratio of spleen weight to body weight revealed that mice infected with Δ*btpB* presented near-normal spleen sizes starting at 4 weeks post-infection (wpi), whereas A19 infection caused persistent splenomegaly (**Figure 2B**). Comparative analysis of the splenic bacterial loads demonstrated that Δ*btpB* colonization in the spleen was significantly lower than that of A19 from 2 wpi onward (**Figure 2C**). Both strains peaked in the splenic bacterial load at 2 wpi, followed by a gradual decline. However, Δ*btpB* was cleared more rapidly, becoming undetectable in the spleen by 6 wpi, while residual A19 persisted at this time point.

Statistical analysis of mouse body weight revealed that, compared with the control group, Δ*btpB* did not significantly induce mouse body weight gain, whereas A19 significantly slowed the rate of mouse body weight gain (**Figure 2D**).

Histopathological examination of the spleen and liver tissues further highlighted the differences. In the spleen, A19 infection caused structural disorganization of the splenic corpuscles, fibrous tissue hyperplasia, and lymphocyte necrosis. In contrast, the Δ*btpB*-infected spleens retained relatively intact corpuscle structures but displayed fibrosis and inflammatory cell infiltration (**Figure 2E**). Overall, Δ*btpB* was not markedly different from A19 in terms of the induction of pathological changes in the host. The Δ*btpB* showed no significant difference from A19 in terms of inducing splenic pathological changes in the host. In the liver, both strains induced comparable histopathological changes, except for transient lymphocyte proliferation which was observed in the Δ*btpB*-infected mice at 1 wpi (**Figure 2F**).

### 3.3 The *btpB* mutant strain had elevated antibody levels in mouse serum

**Figure 3:**
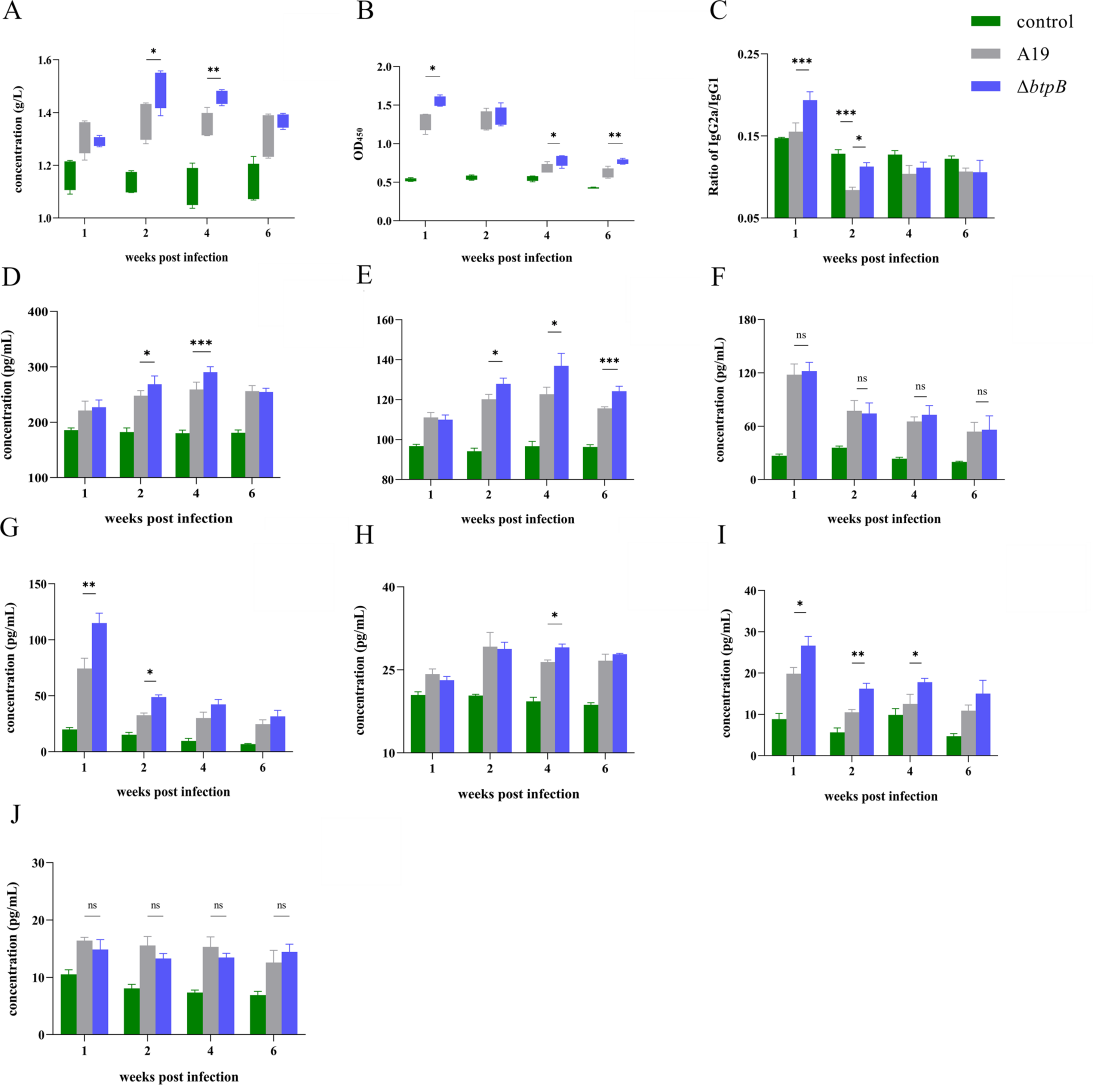
Analysis of antibody and cytokine levels in the serum. **(A)** Total IgG levels in the serum at 1, 2, 4, and 6 wpi. **(B)***Brucella*-specific IgG levels in the serum at 1, 2, 4, and 6 wpi. **(C)** IgG2a/IgG1 ratio in the serum at 1, 2, 4, and 6 wpi. **(D)** IFN-γ levels in the serum at 1, 2, 4, and 6 wpi. **(E)** TNF-α levels in the serum at 1, 2, 4, and 6 wpi. **(F)** IL-12 levels in the serum at 1, 2, 4, and 6 wpi. **(G)** IL-2 levels in the serum at 1, 2, 4, and 6 wpi. **(H)** IL-4 levels in the serum at 1, 2, 4, and 6 wpi. **(I)** IL-1β levels in the serum at 1, 2, 4, and 6 wpi. **(J)** IL-10 levels in the serum at 1, 2, 4, and 6 wpi. All the data are represented as the mean ± SEMs of at least four independent experiments. *p < 0.05; **p < 0.01; ***p < 0.001; ns(not signicant) p>0.05.

ELISA analysis of serum collected at multiple time points revealed that mice infected with Δ*btpB* exhibited significantly higher total antibody levels and *Brucella*-specific IgG levels compared to the A19-infected group at several time points (**Figures 3A, B**), indicating enhanced humoral immunity. Furthermore, the IgG2a/IgG1 ratio, a key indicator for assessing immune polarization, was significantly higher in the Δ*btpB*-infected mice than in the A19-infected mice during the first 2 wpi. Notably, the IgG2a/IgG1 ratio in A19-infected mice was even lower than that in the PBS control group at 2 wpi (**Figure 3C**). These findings collectively demonstrate that Δ*btpB* elicits a stronger humoral immune response in the host.

### 3.4 The *btpB* mutant strain upregulated the level of multiple serum cytokines

To assess Th1 and Th2 immune responses post infection, we measured the levels of Th1-associated cytokines (IL-12, IL-2, IFN-γ, and TNF-α) and Th2-associated cytokines (IL-4, IL-10). The serum IL-12 levels showed no significant changes between the groups (**Figure 3F**). However, compared with the A19 group, Δ*btpB*-infected mice presented significantly elevated IFN-γ, TNF-α, and IL-2 levels at multiple time points compared to the A19 group (**Figures 3D, E, G**). For Th2 responses, IL-4 levels remained largely unchanged, with only a slight increase observed in the Δ*btpB* group at 4 wpi (**Figure 3H**). IL-10 levels were not significantly different between the groups (**Figure 3J**). Additionally, the proinflammatory cytokine IL-1β was universally elevated in the Δ*btpB*-infected mice (**Figure 3I**). These findings indicate that Δ*btpB* enhances Th1-polarized immune responses but has no significantly effect on Th2 responses.

### 3.5 The *btpB* mutant strain induced early CD8^+^ T-cell expansion and enhanced cytotoxic activity during infection

**Figure 4:**
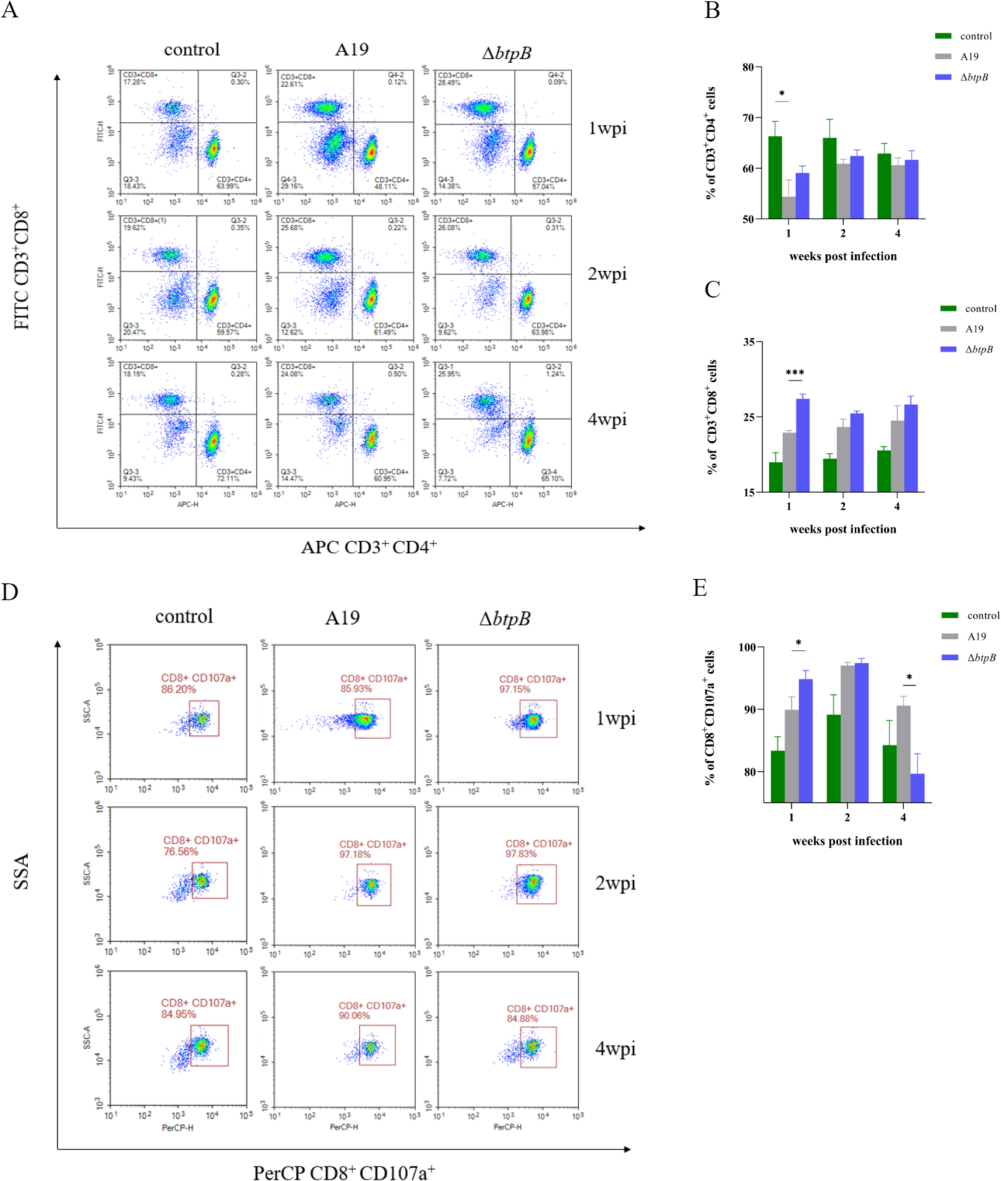
Proliferation and activation of CD4^+^ and CD8^+^ T lymphocytes in mouse spleens at 1, 2, and 4 wpi. **(A)** Flow cytometry analysis of CD4^+^ and CD8^+^ T lymphocytes in the spleen at 1, 2, and 4 wpi. **(B)** Proportion of CD8^+^ T lymphocytes in the spleen at 1, 2, and 4 wpi. **(C)** Proportion of CD4^+^ T cells in the spleen at 1, 2, and 4 wpi. **(D)** Flow cytometry analysis of CD8^+^CD107a^+^ T lymphocytes in the spleen at 1, 2, and 4 wpi. **(E)** Proportion of CD8^+^CD107a^+^ T cells in the spleen at 1, 2, and 4 wpi. All the data are represented as the mean ± SEMs of at least four independent experiments. *p < 0.05; ***p < 0.001.

We used flow cytometry to evaluate the population of CD4^+^ and CD8^+^ T lymphocytes across time points. The results revealed no significant differences in CD4^+^ T-cells between A19 and Δ*btpB*-infected groups. Notably, however, the A19-infected group presented lower proportions of CD4^+^ T cells than the PBS control group at 1 wpi (**Figures 4A, C**). Compared to the A19 group (∼23%), the Δ*btpB* group presented a marked increase in the proportion of CD8^+^ T lymphocytes (>28%) at 1 wpi, although this difference diminished by 2 and 4 wpi (**Figures 4A, B**). Concurrently, the proportion of degranulating CD8^+^ T lymphocytes (CD8^+^CD107a^+^) was significantly greater in the Δ*btpB* group than in the A19 group at 1 wpi but declined below A19 levels by 4 wpi (**Figures 4D, E**). These results suggest that Δ*btpB* triggers a stronger early cytotoxic T lymphocyte response, indicative of enhanced cell-mediated immunity during the initial phase of infection.

### 3.6 The *btpB* mutant strain elicited stronger Th1 and Th2 responses during early infection

**Figure 5:**
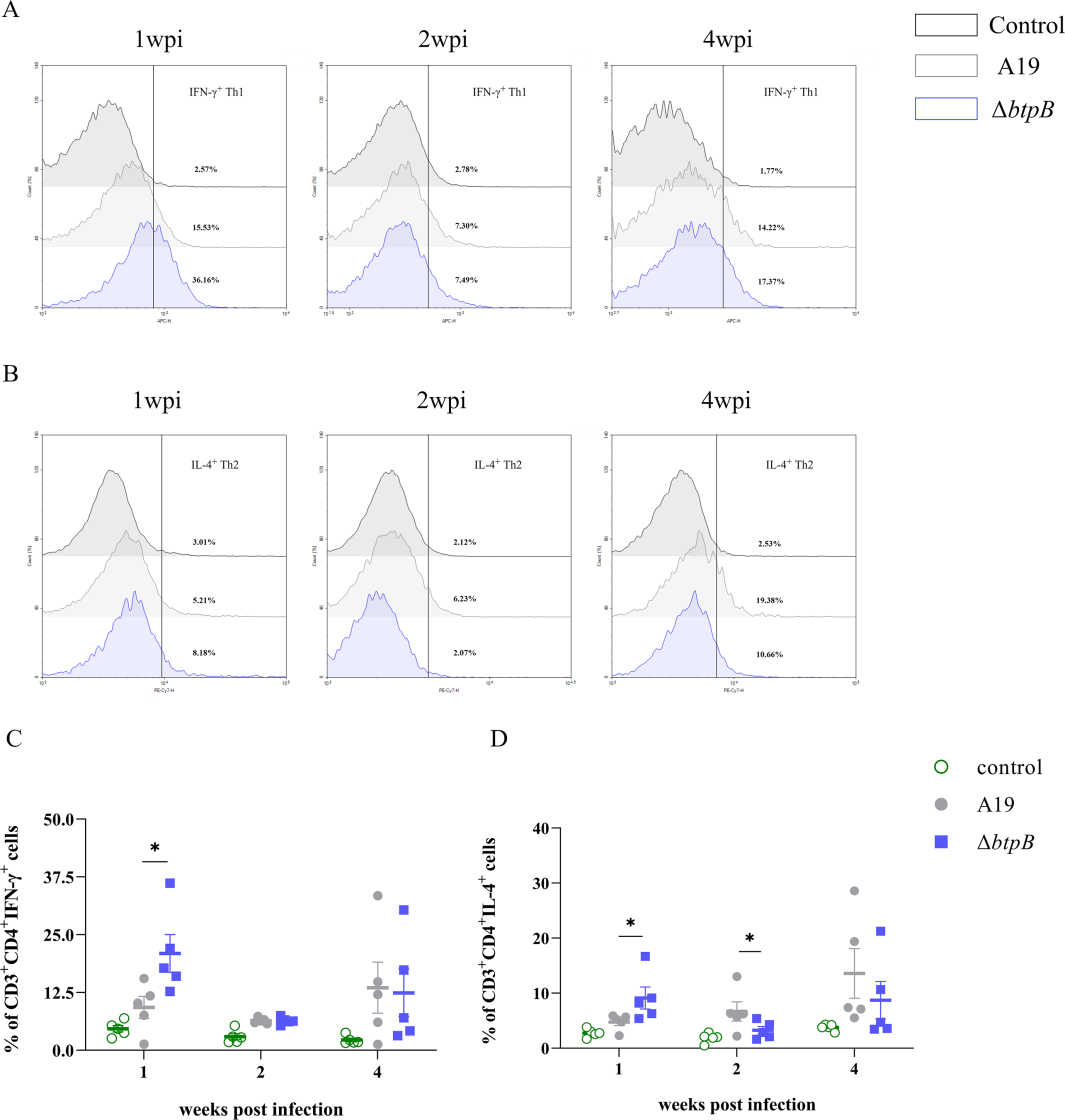
Changes in splenic Th1 and Th2 lymphocytes at 1, 2, and 4 wpi. **(A, C)** Proliferation of Th1 lymphocytes in the spleen at 1, 2, and 4 wpi. **(B, D)** Proliferation of Th2 lymphocytes in the spleen at 1, 2, and 4 wpi. All the data are represented as the mean ± SEMs of at least five independent experiments. *p < 0.05.

To investigate Th1 and Th2 lymphocyte proliferation in the spleen post infection, we performed flow cytometry analysis. The Δ*btpB*-infected mice exhibited significantly higher proportions of Th1 lymphocytes compared to the A19-infected group at 1 wpi (**Figures 5A, B**), consistent with the CD8^+^ T-cells trend (**Figure 4C**). In contrast, the Th2 lymphocyte proportions in the Δ*btpB* group were elevated at 1 wpi but declined below the A19 level by 2 wpi (*p* < 0.05) (**Figures 5C, D**). These results suggest that Δ*btpB* triggers stronger Th1 responses and transiently enhances Th2 responses during early infection, with Th2 activity diminishing thereafter. Notably, both Th1 and Th2 cell proportions decreased significantly at 2 wpi compared with those at 1 wpi but rebounded by 4 wpi, a dynamic that may correlate with bacterial load-dependent immunosuppressive effects.

### 3.7 The *btpB* mutant strain has no influence on the NK cell proportion and elicits stronger NK cell cytotoxicity during mid-to-late infection

**Figure 6:**
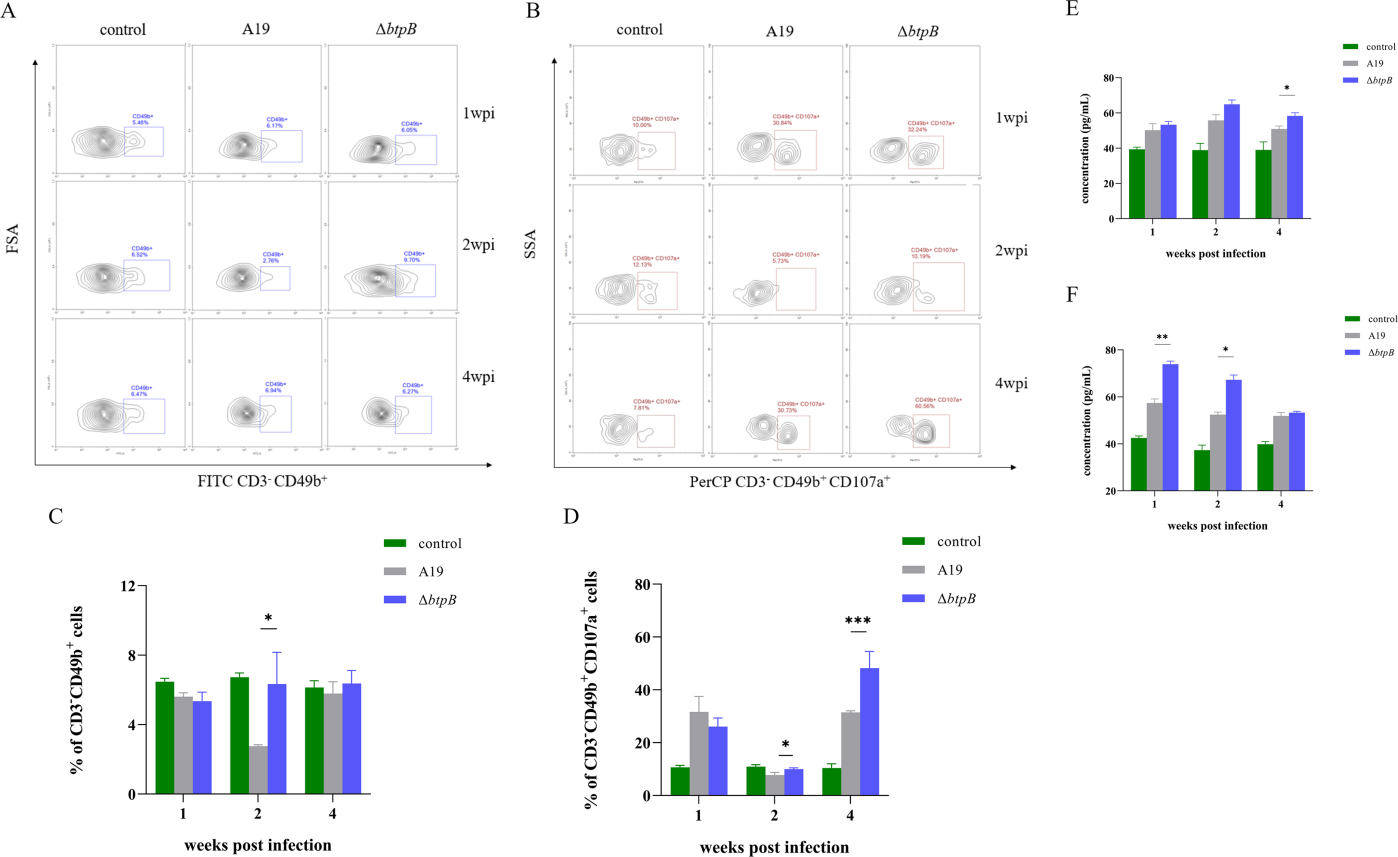
Changes in splenic NK cell proportions and cytotoxic activity at 1, 2, and 4 wpi. **(A, C)** Changes in the proportions of NK cells in the spleen at 1, 2, and 4 wpi. **(B, D)** Changes in the proportions of NK CD107a^+^ cells (indicative of degranulation) in the spleen at 1, 2, and 4 wpi. **(E)** The content of IL-15 in the supernatant of splenocyte cultures. **(F)** The content of IL-18 in the supernatant of splenocyte cultures. All the data are represented as the mean ± SEMs of at least four independent experiments. *p < 0.05; **p < 0.01; ***p < 0.001.

Flow cytometry analysis of splenic NK cell (CD3^-^CD49b^+^) proliferation revealed that, compared with the PBS control group, Δ*btpB* infection caused no significant changes in the proportions of NK cells throughout the 1–4 wpi period. In contrast, the A19-infected group presented a significant reduction in the proportions of NK cells at 2 wpi, which normalized by 4 wpi (**Figures 6A, C**). Concurrently, assessment of NK cell degranulation (CD3^-^CD49b^+^CD107a^+^) demonstrated that Δ*btpB*-infected mice maintained significantly greater cytotoxic activity than did the A19 group at both 2 and 4 wpi (**Figures 6B, D**). We subsequently measured the levels of two key cytokines, IL-15 and IL-18, which are associated with NK cell activation, in splenocyte culture supernatants. The results revealed that the levels of both IL-15 and IL-18 were significantly elevated at various time points at 4 wpi in the Δ*btpB*-infected group. These findings collectively indicate that Δ*btpB* preserves NK cell populations while enhancing their cytotoxic function during later infection stages.

## 4 Discussion

As a key effector protein of the *Brucella* T4SS, BtpB has been demonstrated to function as an NAD^+^ hydrolase. This activity depletes intracellular NAD^+^/NADH pools in macrophages, disrupts host energy homeostasis, and may thereby influence macrophage polarization (25, 26). Furthermore, BtpB suppresses the expression of TLR2 and TLR4 and attenuates NLRP3 inflammasome activation (28). Collectively, these strategies enable *Brucella* to establish a chronic infection within macrophages by evading host clearance, underscoring the critical role of BtpB in immune evasion (28, 29). In this study, the markerless mutant strain Δ*btpB* was successfully constructed via homologous recombination. Assessments of its growth characteristics and *in vitro* stress resistance revealed no differences in growth kinetics compared with A19 but demonstrated weakened stress. Furthermore, using a murine immunization model, we systematically evaluated the *in vivo* colonization capacity, pathogenicity, and immunogenicity of Δ*btpB*. The results demonstrated that BtpB deletion did not significantly affect *Brucella* A19-induced host damage but compromised its survival *in vivo*, which was mediated by eliciting a stronger Th1-polarized immune response. Additionally, analysis of NK cell numbers and cytotoxic activity suggested that BtpB may suppress NK cell cytotoxic function. These findings collectively indicate that Δ*btpB* induced an enhanced immune response, leading to accelerated bacterial clearance. This property of elevated immunogenicity highlights its potential as a live-attenuated *Brucella* vaccine candidate.

*In vitro* and cell-based assays demonstrated that Δ*btpB* retained normal viability, with no significant impairment in its adhesion and proliferation capabilities in macrophages (**Figures 1G, H, D**). However, in the mouse infection models, Δ*btpB* was cleared more rapidly from the spleen (**Figure 2C**). Upon infection with *Brucella*, the host mobilizes a comprehensive immune response to eliminate the bacteria (10, 20). Macrophages serve as a crucial first line of defense against intracellular pathogens such as *Brucella* (30). Although, the adhesion and proliferation capabilities of Δ*btpB* were not different from those of A19 after infecting macrophages, previous studies have confirmed that BtpB inhibits the expression of TLR2 and TLR4 in macrophages, thereby suppressing the host immune response during the early stages of *Brucella* infection (28, 29). Therefore, it is reasonable to speculate that macrophages, as intermediate component, play a role in relaying signals throughout the immune system. Despite the ability of Δ*btpB* to evade macrophage killing as effectively as the wild-type strain does, the altered signals transmitted by macrophages may enhance the activation of other immune cells, leading to accelerated bacterial clearance *in vivo*. Our subsequent research has validated this hypothesis.

This study investigated the multifaceted changes in immune responses elicited by the *btpB* mutant strain, including the comprehensive upregulation of Th1 responses and cell-mediated immunity, which are the key mechanisms of the host to defend against intracellular pathogens such as *Brucella* (12, 31, 32). The IFN-γ level has been demonstrated to be a critical indicator for evaluating the host’s capacity to clear *Brucella*, while IL-2 serves as a key factor mediating Th1 cell differentiation (12, 33, 34). Serum detection revealed that Δ*btpB* induced elevated levels of IFN-γ and IL-2. Since IFN-γ can be secreted by multiple cell types *in vivo*, we further investigated whether the increased serum IFN-γ levels in Δ*btpB*-infected mice were attributed to increased lymphocyte secretion. Previous studies have highlighted the critical role of CD4^+^IFN-γ^+^ cells in controlling *Brucella* infection (12). The results revealed an increased proportion of IFN-γ-secreting Th1 cells in the spleen at 1 wpi. As a major immune organ, the spleen releases large quantities of immune factors into systemic circulation upon pathogen invasion (21, 30), which explains the rapid rise in serum IFN-γ levels observed in Δ*btpB*-infected mice from 2 wpi, surpassing those in the A19-infected group. In addition, we observed rapid proliferation of CD8^+^ T cells and elevated degranulation levels at 1 wpi (**Figures 4B, E**), which explains the accelerated clearance of Δ*btpB* in the spleen. Additionally, IL-12 is known to drive naïve T-cell differentiation into Th1 cells, serving as a critical upstream stimulator of Th1 responses (35). However, the serum IL-12 levels did no differ between the Δ*btpB*- and A19-infected groups, suggesting that the upregulated IFN-γ levels in the Δ*btpB* infection may be mediated through IL-12-independent pathways.

We also observed partial enhancement of humoral immunity, as evidenced by elevated antibody levels, IL-4 levels, and splenic Th2 cell proliferation (36). Δ*btpB* infection induced increased total antibody and *Brucella*-specific IgG levels at multiple time points, along with a transient increase in the serum IL-4 (4 wpi, *p* < 0.05). These findings, however, contradict the reduced proportion of CD4^+^IL-4^+^ lymphocytes in the Δ*btpB* group compared with the A19 group at 2 wpi (**Figures 4H**, **5D**). Given the stronger cell-mediated immunity induced by Δ*btpB*, as we discussed earlier, the decline in CD4^+^IL-4^+^ cells at 2 wpi may reflect a shift toward Th1-dominant immune polarization during infection. Furthermore, Th2 immune responses have been shown to play a limited role in controlling *Brucella* replication (12, 37, 38), which explains why the reduction in CD4^+^IL-4^+^ cell proportions did not hinder the accelerated clearance of Δ*btpB*. However, whether this shift induces other specific immunological changes requires further investigation.

NK cells have been demonstrated to play a critical role in controlling *Brucella* infection (39). *Brucella* infection activates NK cells, which can directly kill infected target cells and secrete IFN-γ and TNF-α to enhance adaptive immune responses by promoting T and B-cell activation (40). However, accumulating evidence suggests that *Brucella* invasion also induces NK cell suppression and immune exhaustion, manifesting as reduced NK cell numbers and impaired cytotoxic activity (21, 41). Therefore, we assessed the proportions of splenic NK cells and their cytotoxic activity at 1, 2, and 4 wpi. Our results revealed that even the attenuated vaccine strain A19 caused a significant decline in the number of NK cells at 2 wpi. In contrast, Δ*btpB* infection did not reduce the number of the NK cells at any time point. Furthermore, analysis of NK cell cytotoxic activity (CD107a^+^) revealed a decline at 2 wpi in both groups, but Δ*btpB*-infected mice maintained marginally higher degranulation levels than the A19 group (*p* < 0.05). The trend persisted and became more pronounced at 4 wpi (*p* < 0.001). In addition, the measurement of IL-15 and IL-18 in the splenocyte culture supernatant also presented that, compared with A19, Δ*btpB* has a significantly greater capacity to activate splenic NK cells (**Figures 6E, F**).

The immunosuppressive effects of *Brucella* on various host immune cells have been extensively studied (20, 21, 36). However, immunosuppression induced by vaccine strains such as A19 has rarely been reported. In this study, multiple indicators demonstrated that A19 infection induced immunosuppression in mice, including reduced CD4^+^ T-cell proportions during early infection (**Figure 4E**), which is consistent with observations of human brucellosis (36), and decreased in Th1, Th2, and NK cell numbers and activity at 2 wpi (**Figures 5C, D**, **Figure 6C, D**). The peak bacterial colonization at 2 wpi (**Figure 2B**) likely explains the observed reduction in immune cell numbers during this period. By 4 wpi, most immune parameters had rebounded to levels comparable to or exceeding those in the PBS control group. This recovery coincided with a rapid decline in the splenic bacterial load (**Figure 2B**). Nevertheless, Δ*btpB* induced weaker immunosuppressive effects than A19 did on certain parameters (**Figures 4E**, **6C, D**), indicating its superior immunogenicity as a vaccine candidate.

During long-term evolution, *Brucella* has developed various immune evasion mechanisms that target multiple immune cells of the host organism (20). The aforementioned study demonstrated that BtpB plays a critical role in the immune evasion process of *Brucella*. Consequently, A19-Δ*btpB* triggered a comprehensive and robust immune response in mice, leading to its rapid clearance within the host. The existing A19 vaccine strain still exhibits residual virulence and has the potential to persist in the host for extended periods, leading to persister formation (42). This represents an obvious drawback for a widely used live attenuated vaccine on a global scale. Therefore, in this study, Δ*btpB* demonstrated promising characteristics as a novel vaccine candidate. Additionally, given the significant immune evasion function of BtpB, targeting it for therapeutic interventions could also be a highly promising research direction.

In summary, the results indicate that, compared with A19, Δ*btpB* has a reduced survival ability in the host and triggers a stronger cellular and humoral immune response. These findings demonstrate that BtpB serves as a crucial immunosuppressive factor in *Brucella*, playing a key role in immune evasion. Its deletion significantly enhances the host’s immune response, suggesting that it can serve as an ideal candidate for developing live-attenuated vaccines. Moreover, BtpB could be targeted to develop novel therapeutic strategies for animal and human brucellosis.

## Data Availability

The original contributions presented in the study are included in the article/supplementary material. Further inquiries can be directed to the corresponding authors.
