# Supplementary material for: Enhanced immunogenicity of the Brucella A19 ΔbtpB mutant leads to accelerated clearance in the host
Source: Front Immunol. 2026 Jan 30;17:1747903. doi: 10.3389/fimmu.2026.1747903 (PMC12901473; doi:10.3389/fimmu.2026.1747903)
Supplement: Supplementary file 1 [file Table1.doc]

| Class | Primer | Sequence（5’-3’） |
| --- | --- | --- |
| PCR | *btpB*-up-F | TGACCATGATTACGCCAAGCTTCTACCTAATCGGCGAGAATCTG |
|  | *btpB*-up-R | CTACCGATTAGACTGGCGGTAATGCATATTCCTCGCTAAGTTCGAT |
|  | *btpB*-down-F | CGAACTTAGCGAGGAATATGCAGTTACCGCCAGTCTAATCGGTAG |
|  | *btpB*-down-R | ATGACTAGTAGATCCTCTAGACAAACGCCATCTCCACCACCAATCT |
|  | *btpB*-F | ATGTACAATTTATTTGTTTCGGGCTGG |
|  | *btpB*-R | CTAGGTGATGAGGGCGACG |
| qPCR | *btpB*-q-F | AAGTGCGAATCGAGTACGAGT |
|  | *btpB*-q-R | CAGGTTTACGTCTTTGACCGC |

**Supplementary Table 1. Primers in this study**
